# Retrograde improvisations and their iatrogenic complications: a case report of an antegrade femoral nail used as a retrograde construct

**DOI:** 10.1093/jscr/rjag156

**Published:** 2026-03-12

**Authors:** Yaseen Ammar, Hussain Mohammad, Hasan Dashti, Salamah H Ayyad

**Affiliations:** Department of Orthopaedic Surgery, Jaber AlAhmad Hospital, PO Box 2270, Kuwait City, Kuwait; Department of Orthopaedic Surgery, Jaber AlAhmad Hospital, PO Box 2270, Kuwait City, Kuwait; Department of Orthopaedic Surgery, Jaber AlAhmad Hospital, PO Box 2270, Kuwait City, Kuwait; Department of Orthopaedic Surgery, Jaber AlAhmad Hospital, PO Box 2270, Kuwait City, Kuwait

**Keywords:** intramedullary nailing, femoral shaft fracture, implant misapplication, hypertrophic non-union, periprosthetic fracture, case report

## Abstract

Intramedullary nailing is the standard of care for femoral shaft fractures. Antegrade nails are designed for proximal insertion, while retrograde nails are for distal insertion, deviation from this pathway can result in mechanical failure. We report a 47-year-old female who presented with left thigh pain, swelling, and restricted mobility. Clinical examination revealed thigh deformity and ecchymosis, with preserved distal neurovascular status. Radiographs demonstrated an intramedullary nail with hypertrophic malunion at the distal femur with a proximal periprosthetic fracture. Computerized tomography imaging confirmed cortical breaches by screws, and an oblique fracture near the proximal screw. Images were suggestive of the use of an antegrade nail through a retrograde construct. The malpositioned nail was removed and replaced using the appropriate proximal entry site. Postoperative radiographs confirmed stable fixation. The patient reported improved mobility with radiographic healing. This case highlights the consequences of incorrect implant orientation and the importance of surgical verification.

## Introduction

Intramedullary nailing remains the preferred method for stabilizing femoral fractures, with entry point selection critical to biomechanical success [1]. Antegrade nails are designed for proximal insertion, and deviations from this orientation can result in serious complications, including non-union and periprosthetic fracture [1]. While deliberate off-label use may be appropriate in select anatomical scenarios, unintentional misuse of implant trajectory is rarely documented [2–4]. This report describes a case of reversed insertion of an antegrade nail, leading to mechanical failure, highlighting the consequences of incorrect implant application.

## Case presentation

A 47-year-old female presented to the Emergency Department after a fall with acute left thigh pain, swelling, and reduced range of motion. She denied constitutional symptoms such as fever, weight loss, or recent infection. Her medical history was significant for a prior cerebrovascular accident and a long ICU admission after a road traffic accident previously.

**Figure 1:**
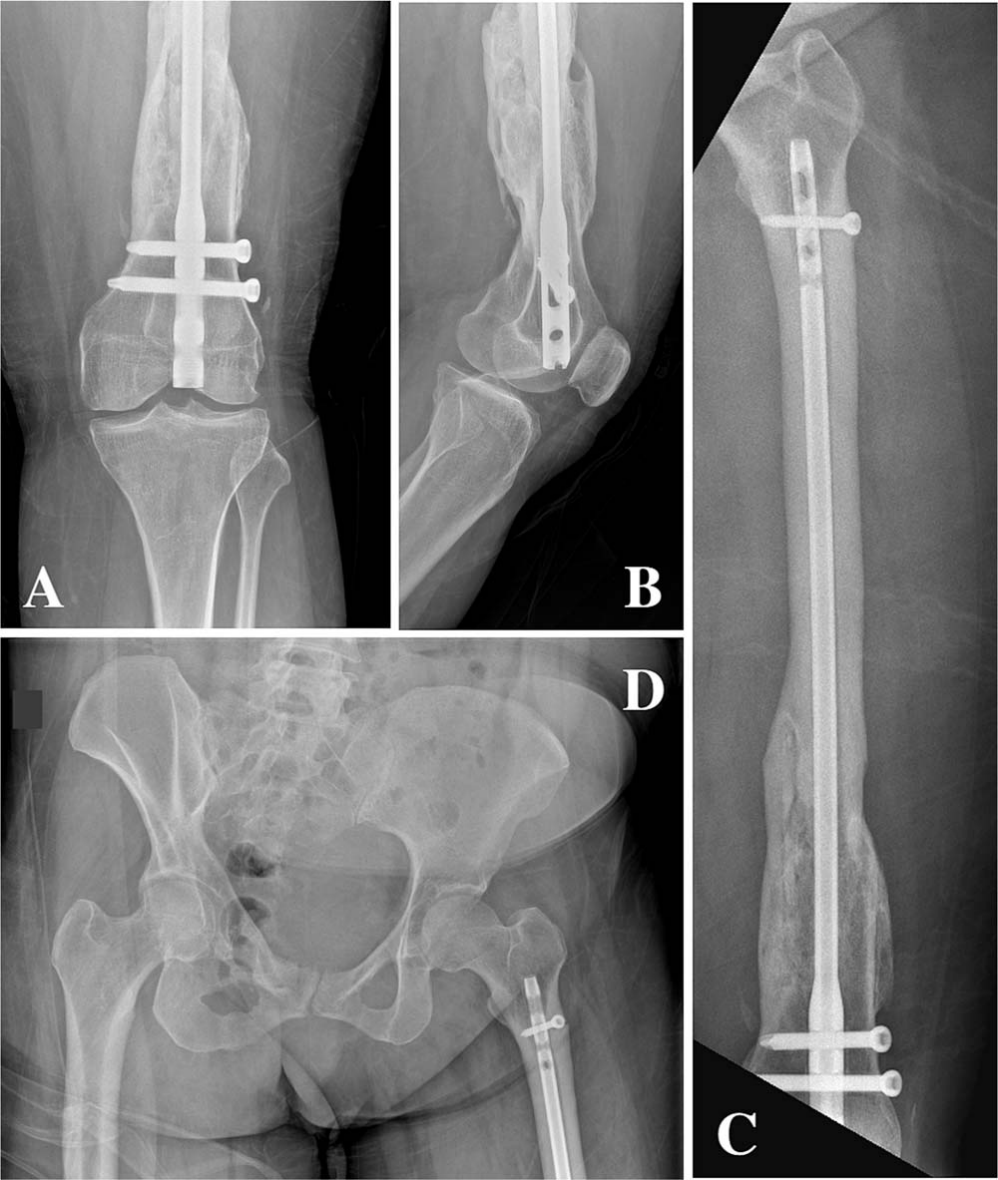
(A, B) Anteroposterior and lateral radiographs showing hypertrophic non-union of the distal femur with retained intramedullary nail and locking screws. (C) Anteroposterior view demonstrating a femoral nail inserted via a distal entry point with abnormal proximal extension. (D) Anteroposterior pelvis radiograph showing the antegrade-design nail extending proximally from a retrograde entry point, terminating proximal to the lesser trochanter.

**Figure 2:**
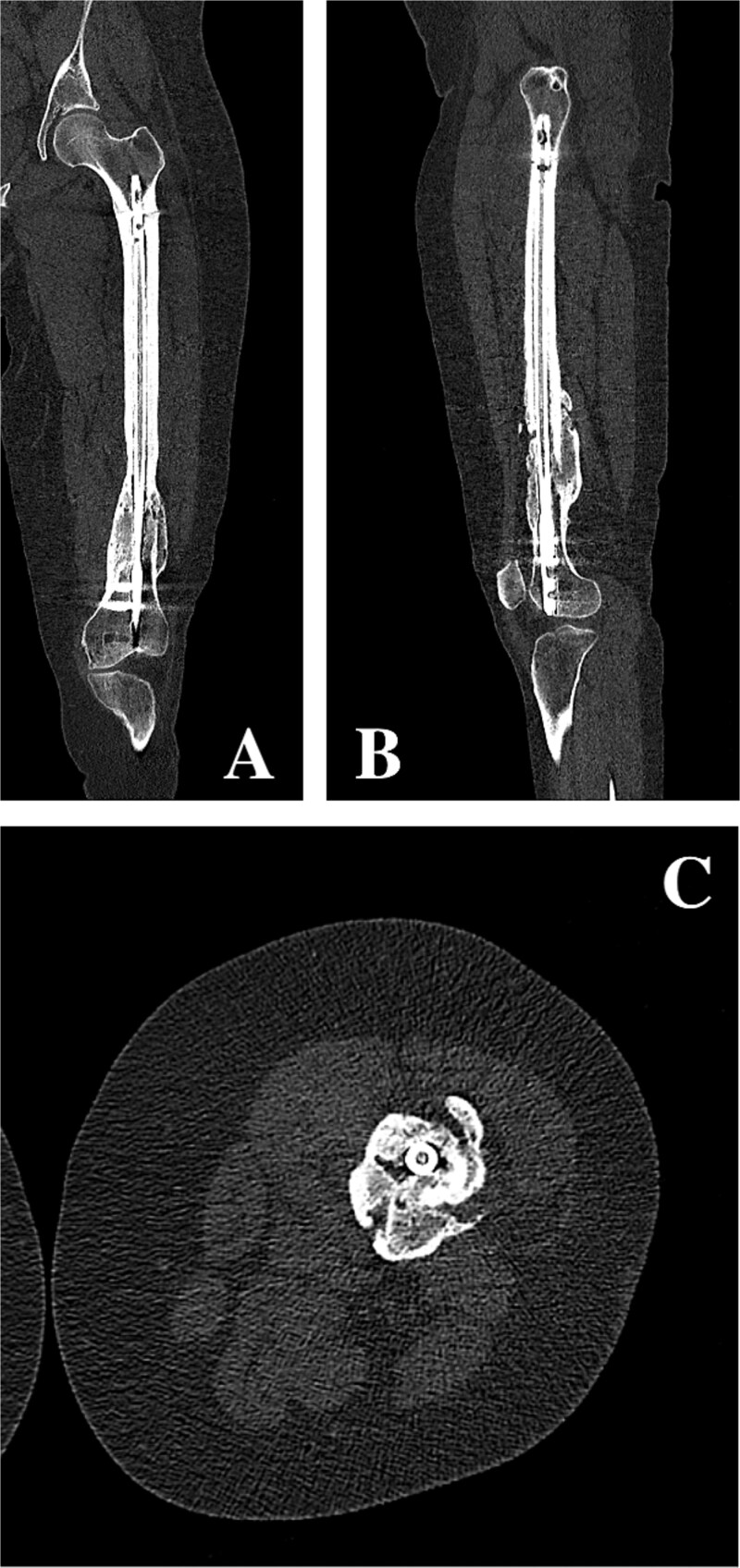
(A, B) Coronal and sagittal CT reconstructions of the left femur demonstrating an intramedullary femoral nail with extensive hypertrophic callus formation at the site of a chronic distal femoral malunion, along with an oblique proximal periprosthetic fracture adjacent to the proximal locking screw. (C) Axial CT image demonstrating hypertrophic callus consistent with a distal femoral malunion surrounding an intramedullary femoral nail.

**Figure 3:**
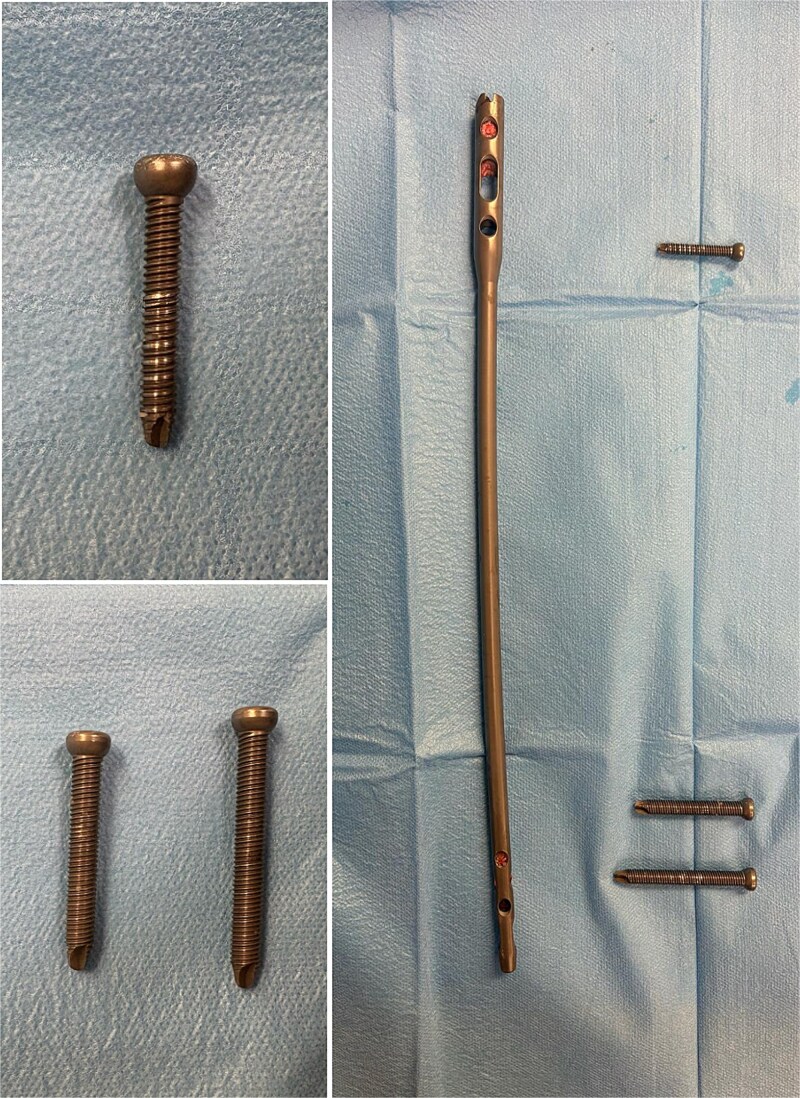
Extracted femoral intramedullary nail and locking screws displayed on a sterile field. The implant’s design confirms it is an antegrade nail, despite its prior insertion through a distal (retrograde) entry point.

**Figure 4:**
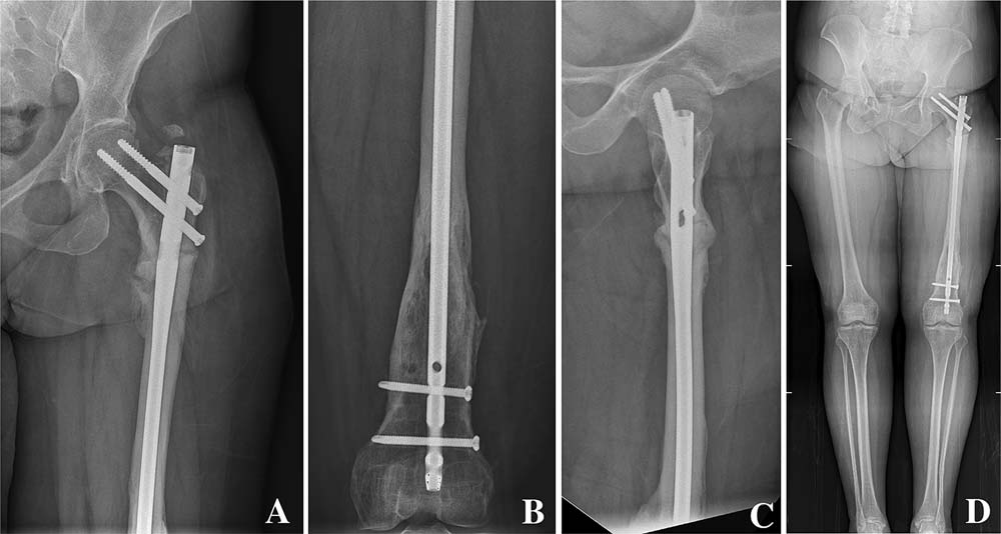
(A, B) Anteroposterior radiographs of the proximal and distal femur showing a correctly positioned antegrade femoral nail inserted via the appropriate proximal entry site. (C) Lateral radiograph of the femur showing stable fixation with appropriate screw placement and no evidence of cortical breach. (D) Scanography of the lower limbs demonstrating restored mechanical alignment.

On clinical examination, the left thigh was swollen and ecchymotic with visible deformity. Passive and active range of motion at the hip and knee was markedly restricted due to pain. Distal neurovascular function was intact. All laboratory investigations were negative or normal. Radiographs of the femur, hip, and knee revealed a hypertrophic malunion at the distal femur with a proximal periprosthetic fracture and an intramedullary nail inserted (Fig. 1). A preoperative non-contrast computerized tomography (CT) scan of the left thigh confirmed an old distal femoral fracture with in situ fixation and extensive hypertrophic callus. Multiple small free bone fragments were seen in the distal thigh. The hardware extended beyond the distal and medial cortical margins, with screws also breaching the cortex. An oblique fracture line was noted proximally adjacent to the proximal screw, without displacement. No soft tissue hematoma or joint effusions were identified (Fig. 2). The patient was admitted for surgical management. Revision antegrade nailing was performed intraoperatively. Upon removal of the retrograde nail it was confirmed to be an antegrade construct (Fig. 3). A correctly sized antegrade femoral nail was inserted via the proximal entry point under fluoroscopic guidance. Postoperative imaging demonstrated satisfactory alignment and stable fixation. At follow-up, the patient reported improved mobility and reduced pain. Radiographs showed signs of progressive healing. No signs of infection, implant loosening, or neurovascular compromise were observed (Fig. 4).

## Discussion

Intramedullary nailing is the cornerstone of femoral shaft fracture fixation, with implants engineered for specific insertion paths, antegrade nails for proximal entry and retrograde nails for distal insertion through the intercondylar notch [1, 5]. Entry point selection is critical for biomechanical alignment, and deviation from implant-specific design can result in complications including cortical impingement, malalignment, and non-union [1, 6, 7]. Nail curvature is vital for alignment; mismatch with anterior femoral bow can cause cortical impingement, malalignment, and potential fixation failure [7].

Simulation-based studies demonstrate that even experienced surgeons frequently misidentify optimal entry points, often placing them anteriorly or laterally, thereby increasing the risk of malpositioning [2]. In Lisitano *et al.’s* multicentre trial, 13.6% of entry points selected for cephalomedullary nailing were anatomically impossible, with anterior misplacement being most common [2]. These findings underscore the complexity of entry point determination and the inadequacy of generalized surgical guides.

Thorough preoperative planning and intraoperative verification are paramount. Ricci *et al.* emphasized that while antegrade nails through the piriformis fossa offer optimal colinear trajectory with the femoral shaft, they are technically demanding and prone to misplacement, especially in obese patients [1]. Moreover, failure to confirm implant type and intended direction can result in reversed insertion and biomechanical failure. As Buseck *et al.* and Patel *et al.* reported, unconventional nail applications must be planned deliberately, with mechanical considerations tailored to abnormal anatomy such as coxa vara [3, 4].

Mechanical complications due to implant misuse are well-documented. The reversed orientation of a femoral nail alters stress distribution, leading to hypertrophic malunion, periprosthetic fracture, or joint surface compromise [2, 8]. Mounasamy *et al.* described subtrochanteric fractures occurring through the proximal end of retrograde nails, attributing failure to stress concentration from malpositioned hardware [9]. Similarly, Krieg *et al.* reported extensor mechanism dysfunction and patella baja following retrograde nailing, highlighting the importance of respecting knee joint anatomy [10].

While rare, deliberate unconventional nail use has been described. Patel *et al.* used a retrograde femoral nail inserted antegrade to treat a subtrochanteric fracture in the setting of significant coxa vara, achieving stable fixation with appropriate screw orientation into the femoral head [4]. Likewise, Buseck *et al.* detailed the off-label use of a humeral nail in a femur with a 98° neck shaft angle, where traditional implants were incompatible. Both cases succeeded because of precise planning and implant adaptation to patient anatomy [3, 4].

In contrast, the current case involved the retrograde use of an antegrade nail, resulting in malunion and periprosthetic fracture, complications consistent with literature describing instability from incorrect implant application [2, 3]. This reinforces the necessity of intraoperative verification and system-level checks, particularly in trauma settings involving trainees or unfamiliar implant systems [11, 12].

This case carries substantial clinical and educational implications. Avoidable complications stemming from implant misuse can be mitigated through rigorous preoperative planning, implant recognition training, and routine verification protocols [2, 11, 12]. Emphasis on implant literacy and system-specific features should be integral to surgical education. Ricci *et al.* advocate that attention to implant orientation, entry trajectory, and patient-specific anatomy directly impacts healing, union rates, and long-term function [1, 13]. Given the increasing diversity of implant systems and anatomical variability, the need for individualized entry strategies and intraoperative vigilance has never been more critical.

## Conclusion

This case illustrates the consequences of implant misapplication in femoral nailing. Reversed insertion of an antegrade nail led to malunion and periprosthetic fracture. Emphasizing implant identification, appropriate entry point selection, and intraoperative verification is essential to avoid preventable technical errors and ensure stable, biomechanically sound fixation.
